# Determination and analysis of harmful components in synthetic running tracks from Chinese primary and middle schools

**DOI:** 10.1038/s41598-019-49142-9

**Published:** 2019-09-04

**Authors:** Xiaoxiao Wu, Ning Li, Hanxu Ji, Haifeng Zhang, Jiangtao Bu, Xiaoming Zhang, Shasha Qian, Yang Yang, Bing Han, Haojie Wang, Ping Ye, Jungui Zhou, Chi Zhang

**Affiliations:** 1 Jiangsu Provincial Supervision & Inspection Center of Green & Degradable Materials, Nanjing Institute of Product Quality Inspection, No. 3 Jialingjiang East Street, 210019 Nanjing, China; 2 Taizhou Institute of Product Quality Inspection, No. 9 Tianhong Road, 225300 Taizhou, China; 3 Key Laboratory of Child Development and Learning Science (Ministry of Education), School of Biological Science & Medical Engineering, Southeast University, No. 2 Sipailou, 210096 Nanjing, China

**Keywords:** Risk factors, Analytical chemistry

## Abstract

In China, incidences involving pupils suffering health problems caused by synthetic running tracks have attracted the public’s attention. However, the existence of known and unknown harmful chemicals in the tracks have not yet been explored. Here, the levels of 16 known harmful ingredients were firstly analyzed in 167 school running tracks. In all samples, the recognized toxic solvents and additives, such as the benzene series, soluble mercury, 3,3′-dichloro-4,4′-diaminodiphenylmethane (MOCA) and toluene diisocyanate monomer (TDI) were under the limits of detection. In contrast, polycyclic aromatic hydrocarbons (PAHs), phthalates, Short chain chlorinated paraffins (SCCPs) soluble lead, cadmium and chromium were found in 86%, 88%, 46%, 81%, 43% and 83% of the specimens, respectively. The levels, toxicology and distribution of these known chemicals were evaluated. Then, a static-headspace gas chromatography-mass spectrometer (GC-MS) method in full scan mode was employed to screen for unknown volatile chemicals. Three groups of chemicals reflecting different kinds of pollution sources were discovered: new solvents, such as N, N-Dimethylformamide, new additives, such as 2-ethylhexanoic acid, and by-products, such as carbon disulfide. In summary, the existence of potential risk factors in school plastic tracks was revealed through exhaustive testing. Moreover, most of the hazardous components detected have been recently included in a new national standard to improve the safety performance of synthetic running tracks.

## Introduction

In China, a number of incidents have been reported nationwide regarding pupils suffering health problems blamed on plastic running tracks from 2015 to 2017^1^. The cases were distributed in nearly 10 provinces, including Jiangsu, Beijing, Henan, Anhui and Shanghai. In these cases, children developed diseases such as nosebleeds, dizzy spells, coughs and even leukopenia associated with the use of problematic running tracks with an “irritating odour”. The reports have attracted much attention from the government and caused great anxiety to the public. Many of the schools involved in the scandal, such as the Beijing No 2 Experimental School, were forced to remove the entire track after students fell ill. Although governmental departments have organized some supervision of and regulations on the quality of the “toxic” tracks, the key hazardous components in the material have not yet been explored.

Before the outbreak of the “poisonous running track” cases, the quality control of running tracks in China was extremely weak. Related regulations for road engineering were used as references in the construction of running tracks, as there were no technical criteria specifically for them. Meanwhile, great problems with the country’s quality standards for rubber materials still exist. The standards for running track products were developed in 1993^2^; they only set restrictions on the use of a few poisonous components, including benzene-class solvents, soluble lead (Pb), cadmium (Cd), chromium (Cr), mercury (Hg) and toluene diisocyanate monomer (TDI), while other potentially harmful components are not included. This lack of inclusion partially explains why most of the plastic running tracks caused damage to pupils’ health but still passed the national quality standards.

The plastic tracks mainly consist of polyurethane (PU), a type of nontoxic material^3–5^. Ethylene propylene diene monomer (EPDM) and other polymers were also used in small ratios in prefabricated tracks^6,7^. Based on the material and production process, the running tracks in China can be divided into the following 2 types: water-permeable and waterproof (including *in situ* cast and prefabricated). The water-permeable plastic track, in which more recycled materials, such as vehicle tire particles, are used to lower the price, is widely used and caused most of the “toxic track” cases. The other type, the waterproof running track, is directly constructed using a two-component PU synthetic-rubber resin slurry, and it contains relatively pure PU. Recently, more waterproof tracks have been newly constructed because of the high concern of the public^8^.

Generally, the PU slurry is prepared from polyether, a chain extender, an MDI/TDI-based polyisocyanate, a plasticizer and fillers. Other solvents and additives can also be added to improve the physical performance and reduce the price. Due to the complexity of the construction process, the harmful substances in the synthetic tracks may be diverse. After a query of existing refs.^9–11^, we determined a panel of possible harmful substances in plastic running tracks. These components include volatiles from solvents and monomers, semi-volatiles from filler oils and by-products, plasticizers, and soluble heavy metals from catalysts. Short-chain chlorinated paraffins (SCCPs)^12^, 3,3′-dichloro-4,4′-diaminodiphenylmethane (MOCA), polycyclic aromatic hydrocarbons (PAHs) and benzene are representative toxins^13^. Other unknown components in addition to those on this panel may also be emitted and influence human health.

Modern chemical analytical techniques have been widely employed to determine the presence of different volatile organic compounds (VOCs) and other harmful components in plastics, related products and the environment^14–17^. Gas chromatography-mass spectrometry (GC-MS) is the most frequently used method to detect VOCs^18^. For example, Llompart *et al*. investigated the presence of hazardous organic chemicals in rubber playgrounds and pavers made from recycled tires^19^. In addition, there were a large amount of reports on the studies of detection and analysis technique for VOCs in tropical waste, schools, museum construction, and so on^20–22^.

Overall, these studies have provided reference methods for the risk analysis of plastic running tracks.

The determination of potentially harmful chemicals will be the basis of uncovering the key causes of “toxic tracks”. To date, no data have been reported apart from the test results of the current standard. Only when sufficient information on the hazardous factors has been accumulated can a subsequent risk assessment be performed^23^. In this study, we tried to collect an exhaustive list of the harmful chemicals that had been mentioned regarding the plastic running track and developed a series of testing methodologies. All data were rendered for analysis to reveal the existence and levels of harmful chemicals in different track types and sources. Furthermore, the static-headspace GC-MS method using scan mode was applied to screen for more unknown volatile compounds in the samples, which could give valuable clues for the discovery of new risk factors.

## Results

### Determination of hazardous chemicals in plastic tracks

Based on the detailed investigation of the construction process, we listed a set of substances to be tested that could be the risk factors of “toxic” running tracks. Table 1 summarizes the tested items with their sources, potential toxicology, isolation techniques and detection methods. The determination results of these items in the running tracks are shown in Table 2. The levels of benzene, toluene, xylenes, TDI, Hg and MOCA were all lower than the limits of detection (LODs) of the corresponding methods. In contrast, the volatile compounds, phthalates, PAHs, Pb, Cr, Cd and SCCPs were determined in 43% to 100% of all samples. In addition, no release rate of benzene was detected, but the detection rates of formaldehyde and TVOC emissions were 4% and 58%, respectively. More than 10 varieties of PAHs were identified, such as naphthalene, benzo [a] pyrene, benz [a] anthracene and fluoranthene. The levels of various PAHs in synthetic tracks showed great differences: the absolute content of them were from 1 mg/kg to 1058 mg/kg.

**Table 1:** Test items with their source, potential toxicology, isolation techniques and detection methods. GC-FID, gas chromatography-flame ionization detection. ICP-MS, inductively coupled plasma-mass spectrometry. TD-GC-MS, thermal desorption-gas chromatography-mass spectrometry. UV-VIS, ultraviolet-visible.

Test items	Source	Potential toxicology	Isolation techniques	Detection method
Benzene	Solvent	Carcinogenicity, biotoxicity	Ultrasonic extraction	GC-FID
Toluene	Solvent	Mutagenicity, reproductive toxicity	Ultrasonic extraction	GC-FID
Xylenes	Solvent	Respiratory and digestive system damage, central nervous system anaesthesia	Ultrasonic extraction	GC-FID
TDI	Residual monomer	Carcinogenicity, allergenicity	Ultrasonic extraction	GC-FID
Pb	Catalyst, Colorant, fillers	Respiratory system irritation, carcinogenicity, mutagenicity	Extraction with HCl	ICP-MS
Cd	Catalyst, Colorant, fillers	Respiratory system irritation, carcinogenicity	Extraction with HCl	ICP-MS
Cr	Catalyst, Colorant, fillers	Eyes, ears, skin and mucosa system, digestive system damage, carcinogenicity	Extraction with HCl	ICP-MS
Hg	Catalyst, Inorganic fillers	Central nervous system damage, brain damage and death	Extraction with HCl	ICP-MS
Volatile compounds	Solvent	Respiratory system irritation, carcinogenic risk	Oven heating	Weighing method
PAHs	Additive, Recycled rubber	Carcinogenic risk	Ultrasonic extraction	GC-MS
SCCPs	Plasticizer, Flame retardant	Biotoxicity, immune system and reproductive system damage, environmental risk	Ultrasonic extraction and SPE	GC-MS-MS
Phthalates	Plasticizer	Reproductive toxicity, hepatotoxicity, nephrotoxicity	Ultrasonic extraction	GC-MS
MOCA	Vulcanizer	Eyes, skin and mucosa irritation, carcinogenicity	Ultrasonic extraction	GC-MS
Release rate of TVOC	Solvent	Respiratory system irritation, carcinogenic risk	Climate test chamber	TD-GC-MS
Release rate of benzene	Solvent	Respiratory system irritation, carcinogenicity, biotoxicity	Climate test chamber	TD-GC-MS
Release rate of formaldehyde	Additive, Material	Respiratory system irritation, carcinogenicity, reproductive toxicity	Climate test chamber	UV-VIS spectrophotometer

**Table 2:** Results of the determination for test items in plastic tracks.

Test items	*n*	Detection rates (%)	Test results (mean ± SD^a^)	Range (Min–Max)	Medians	LOD	Units
Benzene	167	0	<LOD^b^	—	—	0.02	g/kg
Toluene	167	0	<LOD	—	—	0.02	g/kg
Xylenes	167	0	<LOD	—	—	0.02	g/kg
TDI	167	0	<LOD	—	—	0.1	g/kg
Pb	167	81	11.52 ± 17.02	<LOD–101	6.00	0.01	mg/kg
Cd	167	43	0.13 ± 0.28	<LOD–2	<LOD	0.01	mg/kg
Cr	167	83	0.88 ± 1.29	<LOD–4	0.50	0.01	mg/kg
Hg	167	0	<LOD	—	—	0.1	mg/kg
Volatile compounds	123	100	5.83 ± 5.83	1–48	4	—	g/kg
PAHs	147	86	1126.13 ± 1598.92	<LOD–6321	455	0.1	mg/kg
SCCPs	147	46	2.94 ± 8.11	<LOD–68.4	<LOD	0.02	g/kg
Phthalates	147	88	3.48 ± 5.08	<LOD–26.7	1.46	0.005	g/kg
MOCA	24	0	<LOD	—	—	0.5	g/kg
Release rate of TVOC	26	58	0.44 ± 0.55	<LOD–1.7	0.065	0.005	mg/(m^2^·h)
Release rate of benzene	26	0	<LOD	—	—	0.003	mg/(m^2^·h)
Release rate of formaldehyde	26	4	<LOD	<LOD–0.01^c^	—	0.005	mg/(m^2^·h)

*n*, Sample size; ^a^Standard deviation; ^b^Limit of detection; ^c^Release rate of formaldehyde was detected in only one sample. The data less than LOD were handled as a half of the LOD in the analyses.

### Analysis of hazardous chemicals of different track types and sources

Most of the plastic tracks that caused health problems were of the water-permeable type. As public attention has increased, more waterproof and prefabricated tracks have been constructed. The data comparison of 8 items detected in different track types is provided in Table 3. After justification of data between waterproof and prefabricated tracks using Kolmogorov-Smirnov test, the data didn’t coincide with normal distribution (*p* < 0.05). So, Mann-Whitney U test of nonparametric test was applied for the comparison. As the result, the levels of 7 items in waterproof samples, except volatile compounds, were significantly lower than those in water-permeable ones (*p* < 0.05). According to Chi-square test, the detection rates of these 7 items in waterproof samples were obviously reduced compared to the water-permeable ones (*p* < 0.05).

**Table 3:** The data comparison between the waterproof and water-permeable surfaces.

Test items	Types	*n*	Detection rates (%)	Test results (mean ± SD^a^)	Medians	Range (Min–Max)	Units
Pb	A	33	45**	8.43 ± 15.75*	<LOD	<LOD^b^–38.4	mg/kg
	B	134	91**	12.73 ± 17.31*	7.07	<LOD–101	mg/kg
Cd	A	33	18**	0.03 ± 0.07**	<LOD	<LOD–0.3	mg/kg
	B	134	49**	0.15 ± 0.30**	<LOD	<LOD–2	mg/kg
Cr	A	33	58**	0.32 ± 0.35*	0.2	<LOD–1	mg/kg
	B	134	90**	1.03 ± 1.40*	0.5	<LOD–8	mg/kg
Volatile compounds	A	12	100	4.75 ± 3.04	3	1–11	g/kg
	B	111	100	5.97 ± 6.09	4	1–48	g/kg
PAHs	A	33	39**	344.09 ± 1097.07**	<LOD	<LOD–6007	mg/kg
	B	114	99**	1352.91 ± 1651.67**	666	<LOD–6321	mg/kg
SCCPs	A	33	18*	1.26 ± 3.84**	<LOD	<LOD–15.5	g/kg
	B	114	42*	3.44 ± 8.98**	<LOD	<LOD–68.4	g/kg
Phthalates	A	33	58**	1.67 ± 3.97**	0.05	<LOD–18.0	g/kg
	B	114	95**	4.01 ± 5.25**	2.03	<LOD–26.7	g/kg
Release rate of TVOC	A	19	50**	0.61 ± 0.68**	0.011	<LOD–1.7	mg/(m^2^•h)
	B	7	100**	0.94 ± 0.14**	0.900	<LOD–1.2	mg/(m^2^•h)

A, waterproof surface; B, water-permeable surface; *n*, Sample size; *p < 0.05; **p < 0.01; ^a^ Standard deviation; ^b^Limit of detection. The data less than LOD were handled as a half of the LOD in the analyses.

All of the samples were from 13 different regions of Jiangsu Province. Thus, the test result differences among different regions were also analysed. In Table 4, the mean and standard deviation of Pb, Cd, Cr, PAHs, SCCPs, phthalates and volatile compounds in tracks from different cities are enumerated. The justification analysis by Kolmogorov-Smirnov tests and Homogeneity of Variances showed that the data followed normal distribution (*p* > 0.05) and were homogeneous (*p* < 0.05). Therefore, one-way analysis of variance (ANOVA) and Student-Newman-Keuls test (S-N-K Q-test) were applied to compare the intergroup differences. The data exhibited great diversity among the samples from different regions (*p* < 0.05). For example, using the pairwise comparison on the PAHs concentrations, significant differences existed between Suqian and the other 8 regions (*p* < 0.05).

**Table 4:** Comparison of the levels of 7 harmful substance in running tracks from different regions.

Regions	*n*	Pb	Cd	Cr	PAHs	Volatile compounds	SCCPs	Phthalates
		(mean ± SD^a^)mg/kg				(mean ± SD)g/kg		
Zhenjiang	8	15.83 ± 13.75	0.10 ± 0.12	0.55 ± 0.09	677.25 ± 318.79	2.13 ± 0.78	4.32 ± 5.60	2.28 ± 2.10
Yangzhou	9	8.67 ± 14.19	0.12 ± 0.31	1.92 ± 2.44	2307.45 ± 1916.15	4.84 ± 1.31	2.03 ± 5.35	3.22 ± 2.37
Yancheng	10	4.91 ± 6.27	0.02 ± 0.06	0.75 ± 0.29	1692.30 ± 1911.35	5.00 ± 1.55	13.09 ± 14.38	5.63 ± 8.79
Xuzhou	10	18.14 ± 21.78	0.13 ± 0.07	0.24 ± 0.14	550.80 ± 446.62	5.40 ± 1.80	0.29 ± 0.45	3.57 ± 4.35
Suqian	11	26.64 ± 25.94	0.47 ± 0.42	2.73 ± 1.54	2277.45 ± 1756.08	4.36 ± 0.84	2.80 ± 4.24	3.38 ± 3.24
Wuxi	10	15.10 ± 19.15	0.08 ± 0.03	0.35 ± 0.26	131.80 ± 115.24	11.00 ± 9.10	1.97 ± 3.72	9.88 ± 6.70
Taizhou	12	22.75 ± 25.54	0.19 ± 0.37	2.17 ± 1.95	743.25 ± 561.22	4.33 ± 0.84	4.58 ± 6.70	2.82 ± 3.10
Suzhou	9	4.64 ± 5.07	0.04 ± 0.07	0.73 ± 0.24	366.89 ± 335.80	4.44 ± 1.42	0.22 ± 0.59	2.39 ± 3.44
Nantong	10	1.39 ± 0.67	0.01 ± 0.29	0.69 ± 0.21	3642.50 ± 2328.10	4.60 ± 1.36	2.68 ± 5.55	1.32 ± 1.43
Nanjing	53	7.03 ± 11.95	0.04 ± 0.09	0.42 ± 0.57	292.61 ± 883.29	5.12 ± 3.18	0.38 ± 1.58	2.06 ± 4.72
Lianyungang	10	8.10 ± 6.19	0.14 ± 0.12	0.22 ± 0.12	606.60 ± 489.42	13.70 ± 14.44	0.58 ± 1.61	3.12 ± 2.72
Huai’an	9	23.33 ± 15.30	0.54 ± 0.65	1.11 ± 1.73	1528.67 ± 1123.99	5.49 ± 1.77	3.01 ± 3.03	4.73 ± 5.53
Changzhou	6	9.30 ± 10.36	0.07 ± 0.09	0.50 ± 0.24	2092.5 ± 1877.13	4.67 ± 2.43	11.41 ± 25.49	4.41 ± 6.36

*n*, Sample size; ^a^ Standard deviation; The data less than LOD were handled as a half of the LOD in the analyses.

**Figure 1:**
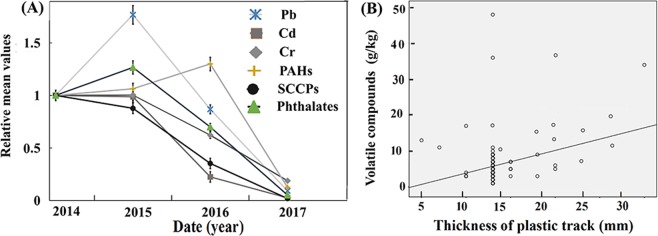
(**A**) Time course of the alteration in the levels of 6 harmful substances from 2014 to 2017. The mean levels of Pb, Cd, Cr, PAHs, SCCPs and phthalates markedly dropped compared with those in the previous year with one-way ANOVA (*p* < 0.05). (**B**) The correlation between the thickness and volatile compound content. A low linear correlation was found between the thickness of the track and the volatile compound content (*r* = 0.23, *p* = 0.01).

Similarly, the harmful compounds in plastic tracks built in different years were compared. The time courses of the main harmful compounds from 2014 to 2017 are shown in Fig. 1A. Results of data justification using Kolmogorov-Smirnov tests (*p* > 0.05) and Homogeneity test for variance (*p* < 0.05) indicated the data were in accordance with normal distribution and homogeneity of variance. So, we used ANOVA analysis and S-N-K Q-test for analysis. Taking the Pb, Cd, Cr, PAHs, SCCPs and phthalates levels in 2014 as references, all of the data markedly dropped compared with those in the previous year (*p* < 0.05). Additionally, the Pb, Cd, Cr, PAHs and phthalates levels showed significant differences from 2014 to 2017 according to the results of one-way ANOVA (*p* < 0.05). In addition, we found a positive correlation between the track thickness and volatile compound contents by correlation analysis (*r* = 0.23, *p* = 0.01, Fig. 1B).

### Screening unknown volatile chemicals in plastic track samples

**Figure 2:**
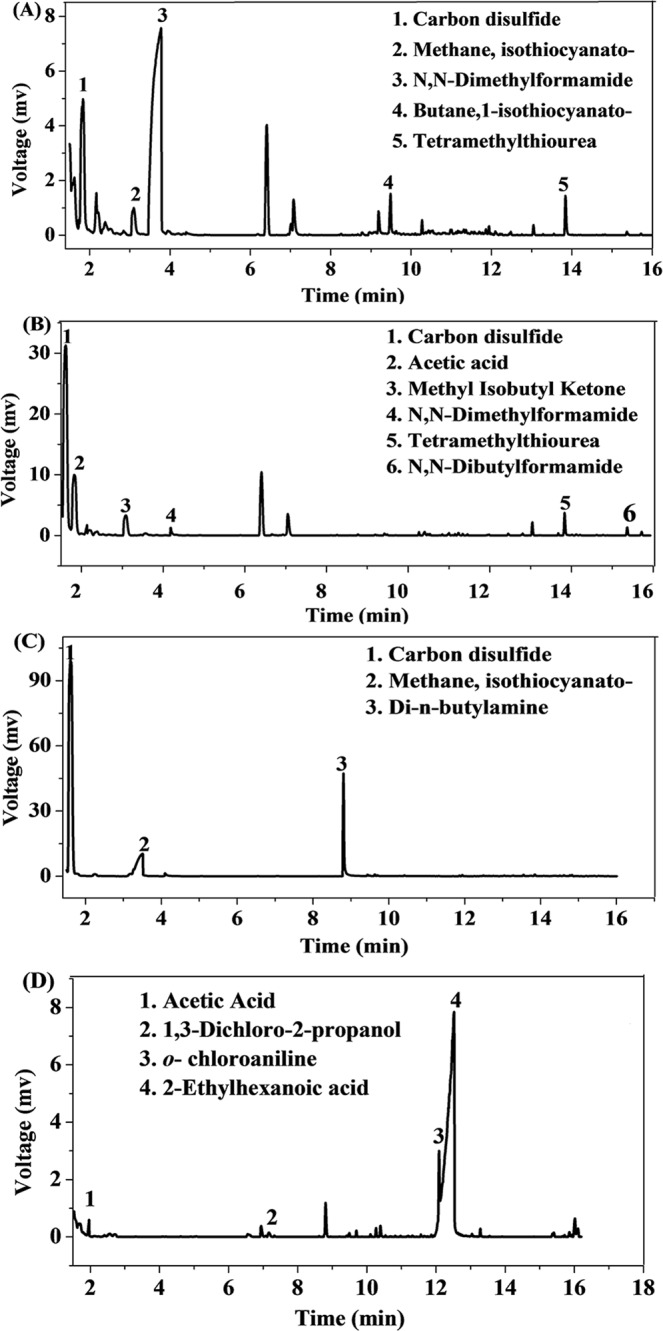
GC-MS chromatograms of 4 representative samples. The chromatograms were obtained by static-headspace GC-MS in full-scan mode. The newly discovered chemicals not on the existing list are labelled in each total ion chromatogram.

To explore more unknown volatile chemicals, 20 plastic track samples with an “irritating odour” were randomly selected. A static-headspace GC-MS approach was used in full-scan mode, coupled with a National Institute of Standards and Technology Library (NIST/EPA/NIH) to discriminate the substances. In each specimen, approximately 6 to 14 chemicals were detected, of which 3 to 6 compounds could be identified. Figure 2 shows the chromatograms of 4 representative samples, and Table 5 lists the newly discovered chemicals not included in the existing list in all of the samples. The 15 identified chemicals can be categorized into three groups: solvents, additives and by-products. The most commonly detected 3 chemicals were carbon disulfide (CS_2_), N, N-Dimethylformamide and 2-ethylhexanoic acid. Their detection frequencies were 85%, 57% and 30%, respectively. Among them, N, N-Dimethylformamide might be a solvent of the polyurethane slurry, 2-ethylhexanoic acid could act as an alternative plasticizer, and CS_2_ came from vulcanization reactions.

**Table 5:** The newly discovered chemicals out of the existing list of risk factors in 20 samples.*The value is the boiling point at 15 mm Hg, while the other values are boiling points at 760 mm Hg (lit.).

Category	Compounds	Molecular formula	CAS	Boiling point [°C]
Solvent	1,3-Dichloro-2-propanol	C_3_H_6_Cl_2_O	96-23-1	174.3
	N,N-Dimethylformamide	C_3_H_7_NO	68-12-2	153
	N,N-Dibutylformamide	C_9_H_19_NO	761-65-9	120*
	Acetic Acid	C_2_H_4_O_2_	64-19-7	117.1
	Acetone	C_3_H_6_O	67-64-1	46.5
	Methyl Isobutyl Ketone	C_6_H_12_O	108-10-1	116.5
Additive	Tetramethylthiourea	C_5_H_12_N_2_S	2782-91-4	245
	2-Ethylhexanoic acid	C_8_H_16_O_2_	149-57-5	228
	Di-*n*-butylamine	C_8_H_19_N	111-92-2	163
	Dimethylamine	C_2_H_7_N	124-40-3	6.1
	Propylene Carbonate	C_4_H_6_O_3_	108-32-7	241.7
	*o*- chloroaniline	C_6_H_6_ClN	95-51-2	208.8
By-product	Carbon disulfide	CS_2_	75-15-0	46.2
	Butane, 1-isothiocyanato-	C_5_H_9_NS	592-82-5	168
	Methane, isothiocyanato-	C_2_H_3_NS	556-61-6	107.8

## Discussion

The tested items in this study are closely related to the pungent odour or potential hazard of synthetic plastic tracks. Among them, organic solvents are necessary to improve the mobility of the PU slurry. As traditional solvents, the benzene series could be directly taken in by the respiratory system, and cause chromosomal aberrations in peripheral blood leukocytes and bone marrow, leading to higher incidences of leukaemia and multiple myeloma after chronic exposure^24^. According to the list of carcinogens published by the World Health Organization’s International Agency for Research on Cancer, benzene is recognized as strong carcinogens^25^. Thus, strict regulations have been set for a long time to limit their use in occupational areas. In the current study, we found the benzene series could not be detected in any of the tested plastic tracks. After consulting the contractors, we learned that the benzene solvents previously used in track construction had been replaced by other organics with a lower toxicity.

The TDI monomers, PAHs, plasticizers, MOCA and soluble heavy metals were also tested, which have been universally accepted as high-concern chemicals that could be absorbed via direct touch. The TDI monomers are irritating and highly allergenic, but they always volatilize and react with water during the protection period (14 days after construction). The samples in the current experiments had been used for at least 6 months, and thus, TDI monomers were not detected. PAHs are important indicators for evaluating the use of recycled rubber and mainly come from the aromatic solvent oil. A high diversity of PAH species and contents was found, indicating the variability of the material quality. Moreover, the recently built waterproof and prefabricated tracks contain much less or no recycled material, and thus, they presented significantly lower PAHs levels compared to the water-permeable tracks. Plasticizers are key additives to make the plastic sufficiently flexible to meet physical standards. However, most studies on “phthalate syndrome” have reported effects on reproductive development in male foetus^26,27^, and SCCPs have also been listed among the substances of very high concern (SVHC) for their adverse effects on the environment and human health. Here, the quantities and percentages of phthalates and SCCPs used in the current running tracks were revealed, and both were closely related to the track types. In our previous tests, MOCA could be occasionally detected in the raw materials of the PU slurry (data not shown); however, it was not found in the finished tracks because of being exhausted in the reaction. Soluble heavy metals, originating from the polymerization catalyst, were measured by inductively coupled plasma mass spectrometry (ICP-MS). Although Hg, Pb and Cr were detected in most of the samples, their absolute concentrations were not sufficient to influence human health.

To determine the overall situation of volatiles in the synthesized tracks, both the volatile compounds and TVOC emissions were determined. The volatile compound method referred to EN 14372-2004 and was performed according to the percentage weight difference between the weight of a sample before and after heating^28^. The TVOC emission test referred to ISO 16000-6-2011^29^, and the TVOC emission was defined as the sum of volatile organic compounds whose boiling points were in the range from (50 °C to 100 °C) to (240 °C to 260 °C), in accordance with the World Health Organization^30^, as determined by the thermal desorption(TD) and GC-MS method^31^. The volatile compounds represented the total content of volatiles, while the TVOC emission reflected the amount of released compounds, thus mimicking the real exposure time and temperature. The mean volatile compound content of all samples was 5.83 g/kg, which is close to the limit for children’s cutlery and feeding utensils in EN 14372-2004 (0.5%). Under the testing conditions of 60 °C for 24 h, TVOC emission was detected in 58% of all tested samples.

Furthermore, we tried to discover more unknown volatile substances using static- headspace GC-MS coupled with a compound library. After *in silico* matching, three groups of chemicals were found: new volatiles in the solvent, new plasticizers and by-products. N,N-Dimethylformamide may act as one component in commercial associative solvents. 2-Ethylhexanoic acid was discovered in prefabricated tracks composed of EPDM, which was most likely a new plasticizer instead of an SCCPs or another plasticizer. The most frequent side-product was CS_2_ from the vulcanization process. Although no recognized chemicals with high acute toxicities were detected, we provide an effective approach for exploring new risky compounds in synthetic running tracks.

In the method design, it was difficult to define and obtain plenty of running tracks with no harmful chemicals as the control group. To optimize the experiment, we authorized a polymer manufacturer to make 4 “pure” materials with minimized addictive that were popularly used in track building: 2 were polyurethane and 2 were EPDM. These 4 extra samples were analyzed for the known and unknown chemicals followed with same methods in this study. The results showed that the levels of all known items were lower than LOD, but some un-evaluated compounds, such as 2-Ethylhexanoic acid and N,N-Dimethylformamide, were also found in the screening test.

During the process of writing this article, the new national standard “Sports areas with synthetic surfaces for primary and middle schools”^32^ was released and will be implemented in October 2018. This new standard has added a number of indices of hazardous compounds with restricted values. Fortunately, that all of the items tested in the current study are included in the new standard with or without minor modifications. In addition, a few other items, such as the benzopyrene content and CS_2_ emission, have been supplemented. The harmful substances in liquid and solid raw materials will also be regulated. Overall, the new standard has greatly raised the bar for the nation’s plastic track construction. Using a group of representative samples from Jiangsu Province, one of the main regions where the “toxic track accidents” occurred, the present study has provided elaborate data for most of the items in the new standard. The data and related analysis could help the industry improve the product formulas and construction process and thus adhere to the new upcoming standard as soon as possible.

As for the determination approaches, classic GC-MS methods were applied to analyse the chemical contents, while the TD-GC-MS method was used to determine the emission levels of the different VOCs. The content and emission values could support each other and reflect the safety status of the samples. Due to the complexity of the harmful compounds, the disease symptoms and pathogenic factors in different incidents can hardly be coincidental. The lack of administration as well as the backward standards led to chaos in the industry, and thus, unexpected toxic chemicals could be added or residual in running tracks. The long-term low level of the industry led to the outbreaks of individual safety cases. Discovering unique pathogenic markers in the sample that actually caused acute poisoning and performing subsequent safety evaluation would also be valuable^8,33^. With the implementation of new standards and the real improvement of the quality, the safety-related cases of plastic tracks will surely be significantly reduced.

## Methods

### Standards

Benzene (≥99.9%); toluene (≥99.9%); xylene (≥99.5%); *n*-tetradecane (≥99.5%); standard solutions of TDI, Pb, Cd, Cr, Hg, MOCA, anthracene-d_10_ and formaldehyde; and standard mixtures of PAHs, SCCPs (55.5% Cl), phthalates and VOCs were provided by Anpel Laboratory Technologies (Shanghai) Inc.

The chemical names of the PAHs, phthalates, and VOCs are as follows: naphthalene, benzo[a]pyrene, benzo[e]pyrene, benz[a]anthracene, benzo[b]fluoranthene, chrysene, phenanthrene, anthracene, acenaphthene, benzo[j]fluoranthene, benzo[g,h,i]perylene, acenaphthylene, benzo[k]fluoranthene, dibenz[a,h]anthracene, fluoranthene, fluorene, indeno[1,2,3-cd]pyrene, and pyrene; di-*n*-butyl ortho-phthalate, butyl benzyl phthalate, bis (2-ethylhexyl) ortho-phthalate, diisononyl ortho-phthalate, diisodecyl ortho-phthalate, and di-*n*-octyl ortho-phthalate; and ethyl acetate, benzene, toluene, xylene, styrene, undecane, and ethylbenzene.

### Reagents and materials

Chromatographically pure ethyl acetate, *n*-hexane, acetone, dichloromethane, and C_18_ solid-phase extraction (SPE) cartridges were purchased from Anpel Laboratory Technologies, Inc. (Shanghai, China).

### Instruments and equipment

A small-scale environmental chamber (100 L, Kunshan Excellence, China), an analytical balance (BSA 224 S, Mettler-Toledo, Switzerland) and a gas chromatography system (7890B, Agilent Technologies, USA) were utilized. A gas chromatography (7890B) and mass spectrometry (5977B) system with electron ionization (Agilent Technologies, USA) was used, and it was equipped with liquid/static-headspace/solid phase micro-extraction autosamplers (PAL RSI 85, CTC Analytics, Switzerland) as well as a NIST/EPA/NIH (Scientific Instrument Services, Gaithersburg, USA). The same model GC-MS system equipped with an automatic thermal desorber (TD) (TD-100, Markes, England) and a 7890 A gas chromatograph with a 7693 automatic sampler interfaced to a 7000 Triple Quad mass spectrometer (GC-QqQ) (Agilent Technologies, USA) were utilized. An ICP-MS system (7900, Agilent Technologies, USA) and an ultraviolet visible (UV-VIS) spectrophotometer (Cary60, Agilent Technologies, USA) were also used.

### Sample collection and preparation

One hundred and sixty-seven samples were collected from primary and middle schools in each city of Jiangsu Province, China. There were 33 waterproof tracks and 134 water-permeable tracks, ranging in thickness from 7 mm to 30 mm. The samples were stored at room temperature.

Moderate amounts of samples were smashed by refrigeration-rubbing, and the tiny particles were used as specimens for the determination of the contents of harmful substances. The plastic tracks were cut into pieces, with a load ratio of 0.4 m^2^/m^3^, at least 20 mm from the sample edge. The test pieces were set aside for 24 h at 60 °C and 5% relative humidity before being placed in small-scale environmental chambers.

### Sample pretreatment and quantitative methods

For the determination of the benzene series and TDI, the specimens (1~2 g) were extracted with ethyl acetate (25 mL) by an ultrasound bath at 60 °C for 1 h. The quantification of the benzene series was performed with external standard (ESTD) method, while TDI was accurately quantified using the internal standard (ISTD) *n*-tetradecane.

Heavy metals were extracted with an acid mixture and kept in contact for 2 h under continuous stirring. The slurry was then filtered and washed, with a detailed description provided in a previous study^34^. Bismuth (Bi), indium (In) and scandium (Sc) were used as ISTDs for Pb/Hg, Cd and Cr, respectively.

The PAHs and SCCPs were extracted with *n*-hexane, while the MOCA and phthalates were extracted with acetone and dichloromethane, respectively. Anthracene-d_10_ was added as an internal standard for MOCA before ultrasonic extraction. The extracted solution of SCCPs was purified and separated by a C_18_ SPE cartridge eluted with 5 mL of a mixed solution of *n*-hexane and dichloromethane (v/v = 1/2). Approximately 2.5 g of each prepared specimen was added into the head space bottle for the detection and recognition of unknown volatiles.

Small-scale environmental chambers were used to simulate the release of harmful substances. The background noise was detected through air sampling after gas exchange was performed six times. The samples were put in the middle of the environmental chamber at 60 °C and 50% relative humidity for 24 h. The gas from the chamber was collected by Tenax absorption tubes to enrich the TVOC, and the formaldehyde was absorbed by MBTH (see GB/T 18204.2 for details^35^). The sampling flow rate was 200 mL·min^−1^, and the sampling volume was 10 L. The ESTD method was used for quantitative analysis.

A weighing method was utilized to evaluate the volatile compound content. The specimen was put in a desiccator for 48 h at room temperature, and approximately 10 g of the sample was added into a container, which was then placed in a drying oven at 200 ± 5 °C with a fresh air inlet for 4 h. The volatile compound content was calculated from the percentage weight difference.

### Instruments, conditions and quantitative methods

Using the standard as a guide^36^, the benzene series and TDI were detected by GC ith flame ionization detection (FID) after being separated by a DB-624 (60 m × 0.25 mm × 0.25 μm) capillary column and an HP-5 (30 m × 0.25 mm × 0.25 μm) capillary column.

The critical instrumental parameters selected for ICP-MS were as follows: RF power, 1300; plasma argon flow rate, 15 L·min^−1^; auxiliary argon flow rate, 1.15 L·min^−1^; carrier argon flow rate, 1.00 L·min^−1^; sampler, nickel; and isotopes monitored, ^208^Pb, ^111^Cd, ^52^Cr, ^201^Hg, ^209^Bi, ^115^In and ^45^Sc.

The PAHs, phthalates and MOCA were detected via a GC-MS method with a liquid autosampler. A capillary column (HP-5MS, 30 m × 0.25 mm × 0.25 μm) was used for separation. The temperature of injection was 280 °C. The electron beam energy setting of the MS was 70 eV. The injection port was operated in splitless mode and equipped with a 1.5 mm inner diameter glass liner. Helium (99.99% pure) was used as the carrier gas at 1 mL·min^−1^ in constant flow mode. The temperature of the ion source was 230 °C for MOCA and phthalates and 270 °C for PAHs. The oven temperature program for phthalates and PAHs was as follows: held at 50 °C for 2 min; increased to 200 °C at 25 °C·min^−1^ and held for 5 min; and then increased to 300 °C at 8 °C·min^−1^ and held for 6 min. For MOCA, the column temperature was initially held at 50 °C for 2 min, increased to 200 °C at a rate of 20 °C·min^−1^, increased to 300 °C at a rate of 8 °C·min^−1^ and then held for 5.5 min. The two qualifier ions and quantifier ion were as follows: MOCA (266, 98, 231) and anthracene-d_10_ (189, 94, 188).

The detection of SCCPs was performed on the GC-QqQ with an HP-5MS (30 m × 0.25 mm × 0.25 μm) column for separation. The oven temperature program was 90 °C (1 min), 25 °C·min^−1^ to 150 °C and 8 °C·min^−1^ to 300 °C (15 min). Other measurement parameters of the instrument were provided in a recent publication^37^.

The static-headspace GC-MS approach in full-scan mode was used to explore the unknown volatiles in plastic track samples. Samples were injected in split mode with a split ratio of 10:1 after thermal treatment at 150 °C for 2 h. The oven temperature program was 40 °C (10 min) and 10 °C·min^−1^ to 300 °C (3 min). The chemical constituents of the detected substances were identified by the NIST/EPA/NIH, with a matching score in excess of 80%.

The release rates of TVOC and benzene were detected by the TD-GC-MS method using a DB-624 (60 m × 0.25 mm × 0.25 μm) capillary column. Helium (99.99% pure) was used as the carrier gas to pre-purge the system for 1 min, with a flow rate of 30 mL·min^−1^. Sorbent tubes were thermally desorbed at 300 °C into the packed liner for 10 min using a flow of inert gas to extract analytes from the samples. The analytes were refocused on a semiconducting cold trap at −10 °C. The trap was quickly heated to 280 °C for 5 min, and the trapped analytes were released and swept through the heated transfer line (at 210 °C) to the GC column. The oven temperature program was 45 °C (1 min), 10 °C·min^−1^ to 70 °C, 20 °C·min^−1^ to 120 °C, 10 °C·min^−1^ to 200 °C and 20 °C·min^−1^ to 220 °C (5 min). Samples were injected in splitless mode. The detection of formaldehyde was accomplished with a UV-VIS spectrophotometer using an absorption wavelength of 630 nm.

### Statistical analysis

All data were analysed by PASW statistics 18.0 (SPSS) for descriptive and correlation analyses. Firstly, Kolmogorov-Smirnov test was used to justify whether the data were normal distribution. The parametric tests would be applied when normality and homogeneity of variance assumptions are satisfied, otherwise the equivalent non-parametric test would be used. The one-way ANOVA was used to compare the difference of measurement data among multiple groups. Pairwise comparison was performed with S-N-K Q-test. Mann-Whitney U test of nonparametric test was used to compare two groups of measuring data with independent samples. Chi-Square test was applied to analyse the detection rates of the test items in waterproof samples and water-permeable samples. Correlational analysis was conducted between track thickness and contents of hazardous materials.

### Capsule

The study include the thorough determination, screening and analysis of harmful substances in synthetic running tracks at Chinese primary and middle schools.

## Data Availability

The authors claim that all the supporting data is available from the authors.
